# CALLR: a semi-supervised cell-type annotation method for single-cell RNA sequencing data

**DOI:** 10.1093/bioinformatics/btab286

**Published:** 2021-07-12

**Authors:** Ziyang Wei, Shuqin Zhang

**Affiliations:** Department of Statistics, University of Chicago, Chicago, IL 60637, USA; School of Mathematical Sciences, Fudan University, Shanghai 200433, China; School of Mathematical Sciences, Fudan University, Shanghai 200433, China; Laboratory of Mathematics for Nonlinear Science, Fudan University, Shanghai 200433, China; Shanghai Key Laboratory for Contemporary Applied Mathematics, Fudan University, Shanghai 200433, China

## Abstract

**Motivation:**

Single-cell RNA sequencing (scRNA-seq) technology has been widely applied to capture the heterogeneity of different cell types within complex tissues. An essential step in scRNA-seq data analysis is the annotation of cell types. Traditional cell-type annotation is mainly clustering the cells first, and then using the aggregated cluster-level expression profiles and the marker genes to label each cluster. Such methods are greatly dependent on the clustering results, which are insufficient for accurate annotation.

**Results:**

In this article, we propose a semi-supervised learning method for cell-type annotation called CALLR. It combines unsupervised learning represented by the graph Laplacian matrix constructed from all the cells and supervised learning using sparse logistic regression. By alternately updating the cell clusters and annotation labels, high annotation accuracy can be achieved. The model is formulated as an optimization problem, and a computationally efficient algorithm is developed to solve it. Experiments on 10 real datasets show that CALLR outperforms the compared (semi-)supervised learning methods, and the popular clustering methods.

**Availability and implementation:**

The implementation of CALLR is available at https://github.com/MathSZhang/CALLR.

**Supplementary information:**

Supplementary data are available at *Bioinformatics* online.

## 1 Introduction

Single-cell RNA sequencing (scRNA-seq) technology has been more and more widely applied in different scenarios in biomedical fields nowadays (Kolodziejczyk *et al.*, 2015; Tang *et al.*, 2009). It measures the gene expressions at each single-cell level. Thus by analyzing the transcriptome-wide cell-to-cell variations, we can study the heterogeneity of different cell types within complex tissues (Kelsey *et al.*, 2017; Liu and Trapnell, 2016; Stubbington *et al.*, 2017), explore the cell-state progression in the developing embryos (Li *et al.*, 2018; Wagner *et al.*, 2018), characterize the diversity of human brain cells (Darmanis *et al.*, 2015; Lake *et al.*, 2018), investigate the heterogeneity of the cancer ecosystems to study the disease progression and response to therapy (Friebel *et al.*, 2020; Tirosh *et al.*, 2016; Wagner *et al.*, 2019; Zheng *et al.*, 2018) and so on. With the fast development of single-cell sequencing platforms, such as Seqwell and 10X chromium3, scRNA-seq data composed of more and more cells are available.

An essential step in scRNA-seq data analysis is the annotation of cell types. Traditional cell-type annotation methods mainly include two steps: clustering the cells using unsupervised learning method, and labeling each cluster manually based on aggregated cluster-level expression profiles and the marker genes (Zhang *et al.*, 2019b). Such methods can be cumbersome, and the accuracy relies on both the clustering accuracy and the prior knowledge on marker gene expression levels. Recently, several cell-type annotation methods using the reference database have been developed. These methods usually map the unannotated cells to the pre-annotated reference datasets using selected features (Aran *et al.*, 2019; de Kanter *et al.*, 2019; Kiselev *et al.*, 2018; Hou *et al.*, 2019; Shao *et al.*, 2020). Then, the cell types are assigned according to the cells’ nearest neighbors or some similarity measures. For example, ‘scCATCH’ selects the marker genes as features, and uses them to map the unannotated cells to the tissue-specific cell taxonomy reference databases to determine the cell types. The performance of such methods depends on the clustering results, and the expression profiles from experiments with different designs may not be directly comparable. Deep learning methods for cell-type annotation have also been proposed (Brbić *et al.*, 2020; Hu *et al.*, 2020). MARS was proposed to project all cells in a meta-dataset into a joint low-dimensional embedding space shared by both annotated and unannotated cells. By learning the cell-type-specific landmarks, it can discover cell types that have never been seen before and annotate experiments that are as yet unannotated (Brbić *et al.*, 2020). ItClust is an iterative transfer learning algorithm with neural network that utilizes external well-annotated source data as the initialization for the target data to better cluster the target cells (Hu *et al.*, 2020). All of these methods integrate the information across multiple datasets. A few automatic cell-type annotation methods were proposed for one single dataset using the marker genes. Zhang *et al.* (2019a) proposed a probabilistic cell-type assignment model ‘CellAssign’ to do the inference, which leverages the prior knowledge of cell-type marker genes to annotate the cells. Another method ‘Garnett’ first labels a number of cells by scoring the marker genes, then uses sparse logistic regression to classify the cells (Pliner *et al.*, 2019).

According to the above analysis, clustering plays leading roles in most cell annotation methods. Though a large number of cell clustering methods have been proposed (Ding *et al.*, 2018; Grun *et al.*, 2016; Ji and Ji, 2016; Huh *et al.*, 2020; Kiselev *et al.*, 2017; Lin *et al.*, 2017; Marco *et al.*, 2014; Ntranos *et al.*, 2016; Park and Zhao, 2018; Pierson and Yau, 2015; Tian *et al.*, 2019; Wang *et al.*, 2017; Yang *et al.*, 2019), they are still not sufficient for accurately annotating the cells. Semi-supervised learning, as a branch of machine learning, uses both labeled and unlabeled data to perform supervised or unsupervised learning tasks (Van Engelen and Hoos, 2020). It has been widely applied in many different fields, including single-cell data analysis (Wu *et al.*, 2020; Zhang *et al.*, 2019c). The advantage of semi-supervised learning is that it can make full use of the prior knowledge on the labeled and unlabeled data, which can lead to better data explanations.

In this article, we present a transductive semi-supervised method called Cell type Annotation using Laplacian and Logistic Regression (CALLR) for annotating the cell types in one single scRNA-seq dataset. Given a dataset consisting of labeled and unlabeled cells with the corresponding subsets denoted as *Z* and *Z*′Z′, we propose a model to produce predicted labels for the unlabeled cells in *Z*′Z′. The model combines the supervised learning part, which uses sparse logistic regression, and the unsupervised learning part, which is represented as a graph Laplacian constructed from all the cells, to learn the unknown cell labels. It is formulated as an optimization problem, and the numerical algorithm for solving it is presented. Here, we suppose a small number of labeled cells are known in the dataset, which may be obtained manually as traditionally do, or learned using marker genes, such as the method developed in Garnett (Pliner *et al.*, 2019). We apply CALLR to several datasets to show its performance. We first compare with some existing clustering methods and (semi-) supervised learning methods. We then show that when a very small proportion of cells are annotated, high annotation accuracy can be achieved. Compared to clustering, higher clustering accuracy can be obtained and cell types can be directly assigned to the clusters at the same time. Compared to supervised learning methods, such as logistic regression, much fewer labeled cells are needed and much higher annotation accuracy is obtained. All these results show the advantages of our proposed method.

## 2 Materials and methods

### 2.1 The CALLR framework

Given an *m *×* n* scRNA-seq gene expression matrix $X=(x_{1},x_{2},\ldots ,x_{n})$ with *m* genes and *n* cells, where *x*_i_$x_{i}$ is the gene expression corresponding to the cell *i*. We first remove the genes with zero expression across all the cells. *X* is then normalized by size factor to adjust for read depth, which is the same as that used in (Pliner *et al.*, 2019). Without confusion, we use *X* as the normalized data matrix. Suppose a small proportion of cells have been annotated, and the cell set is denoted as *Z*. The set of the remaining cells is denoted as *Z*′Z′. We assume the putative number of cell types is given as *K*, which can be seen from *Z*. The cell sets for type *k* is denoted as *C_k_*. Let the cell annotation matrix $U_{K\times n}=(u_{1},u_{2},\ldots ,u_{n})$ be defined as: *u_ki_* = 1 if cell *i* belongs to cluster *C_k_*$i\in C_{k}$, *u_ki_* = 0 otherwise, where $u_{i}$ denotes the annotation vector of the *i*-th cell. Let *g_i_* denote the cell type that the cell *i* belongs to. For each cell *i* in *Z*, the corresponding $u_{g_{i}i}=1$. We build a semi-supervised model to infer the cell types of those in Z′.

CALLR achieves the cell annotation matrix *U* through the following optimization framework:

$$\begin{array}{c} \underset{\alpha ,\beta ,U}{\min}\;-\sum_{i\in Z}\;\text{log}\;Pr(u_{g_{i}i}=1|x_{i})-\sum_{i\in Z^{\prime }}\sum_{k=1}^{K}u_{ki}\;\text{log}\;Pr(g_{i}=k|x_{i})\\ \;+\lambda_{1}\text{tr}(ULU^{T})+\lambda_{2}\sum_{k=1}^{K-1}\left|\right|\beta_{k}|{|}_{1}\\ s.t.\;\;\text{log}\;\frac{Pr(g_{i}=k|x_{i})}{Pr(g_{i}=K|x_{i})}=\alpha_{k}+\beta_{k}^{T}x_{i},\;\forall 1\leq k\leq K-1,\forall i,\\ \;\;u_{ki}=0\;\text{or}\;1,\;\sum_{k=1}^{K}u_{ki}=1,\;\forall i\in Z\prime ,\\ \;\sum_{k=1}^{K}Pr(g_{i}=k|x_{i})=1,\;\forall i. \end{array}$$

Here, *λ*_1_ and *λ*_2_ are non-negative tuning parameters. *L* is the Laplacian matrix corresponding to the adjacency matrix constructed from the gene expression matrix *X*. After obtaining the 0–1 *K *×* n* matrix *U*, the label of each cell *i* is the position where the element in the column vector $u_{i}$ equals to 1.

The intuition of this optimization problem is to combine sparse logistic regression and spectral clustering, which correspond to the supervised and unsupervised part, respectively. The first term in the optimization problem comes from logistic regression for the annotated cells, with *α* being a (K−1)×1 vector explaining the intercept, and β being the coefficient matrix of the *m* genes with size m×(K−1). The third term comes from spectral clustering, which clusters the cells based on their similarities. The second term establishes a connection between them, which tries to make the results of logistic regression and spectral clustering correspond with each other. The fourth term is a regularization penalty term for the coefficients to avoid the overfitting.

Let $P=(P_{1},\;\ldots \;,P_{n})$ be the probability matrix for all the cells being in each cell type, where $P_{i}={\left(Pr(g_{i}=1|x_{i}),\ldots \;,Pr(g_{i}=K|x_{i})\right)}^{T}$. Ideally for each cell *i*, *P_i_* should have one position near 1 and the other positions near 0. So when we calculate $u_{i}$, we expect it to take a larger value (near its maximum 1) if the result from logistic regression and spectral clustering are corresponded. Besides, the second term also utilizes the unlabeled cells in sparse logistic regression. Thus by solving this optimization problem, we expect there is a clear classification of the cells into different types.

### 2.2 Optimization algorithm

The objective function in the optimization problem is nonconvex, but the objective function for logistic regression and spectral clustering are both convex. Thus, we optimize both parts alternately.

Laplacian Matrix . Before the iteration steps, we first need to compute the Laplacian matrix of the gene expression data. We apply *k*-nearest neighbors method to the Euclidean distance to construct the adjacency matrix *A*. We require that cell *j* has a connection to cell *i* if and only if both cell *j* and cell *i* are within their *k*-nearest neighbors, and set *A_ij_* = 1. Otherwise we have *A_ij_* = 0. With this, we have the structure of the similarity graph. Then we use Gaussian kernels to generate the weights for the edges in *A*. For each edge with *A_ij_* = 1, we calculate their similarity *S_ij_* using the same kernel as that in SIMLR (Wang *et al.*, 2017). We set the variance in Gaussian kernel as 1 and set the number of neighbors being 17 as default to get the empirical performance. The results are stable when both parameters take small changes. The Laplacian matrix is computed in the same way as spectral clustering does.

*Initialization*. We run logistic regression on the labeled training data to get the initial *α* and β. Then we predict the labels of the unlabeled cells to get the initial value of matrix *U*.

Step 1: Fix α and β to update U . We rewrite the objective function with respect to (w.r.t) the label matrix *U* as follows:

$$\begin{array}{c} \underset{U}{\min}\;-\mu \sum_{i\in Z^{\prime }}\sum_{k=1}^{K}u_{ki}\;\text{log}\;Pr(g_{i}=k|x_{i})+\text{tr}(ULU^{T})\\ s.t.\;\;u_{ki}=0\;\text{or}\;1,\;\sum_{k=1}^{K}u_{ki}=1,\;\forall i\in Z\prime , \end{array}$$

where we set $\mu =\frac{1}{\lambda_{1}}$ in the original objective function.

Since there are 0–1 constraints in this problem, and the dimension of *U* is quite large, it is inefficient to directly solve such optimization problem using binary optimization methods. We instead develop a projected gradient descent method and a thresholding step to approximate the solution iteratively. We solve the optimization problem by the following two steps.

1. The gradient descent step:

$$\tilde{U}=U^{N}+△t(-U^{N}L+\mu \;\text{log}\;P),$$

2. Projection and thresholding step:

$$u_{i}^{N+1}=\mathrm{projectToVertex}(\mathrm{projectToSimplex}({\tilde{u}}_{i})),$$

where *projectToSimplex* projects a given vector to the simplex, while *projectToVertex* maps a vector to its nearest standard unit vector. Specifically, *projectToSimplex* finds ***w*** for a given vector $v\in R^{K}$, and is defined as:

$$\mathrm{projectToSimplex}(v)=\arg\underset{w\in \Delta^{K}}{\min}||w-v|{|}_{2},$$

where

$$\Delta^{K}:=w={\left(w_{1},\ldots \;,w_{K}\right)}^{T}\in R^{K}:0\leq w_{i}\leq 1,\;\text{and}\;\sum_{i=1}^{K}w_{i}=1,$$

while

$$\mathrm{projectToVertex}(v)=\arg\underset{w\in \Delta^{K}}{\min}||w-v|{|}_{2},$$

where

$$\Delta^{K}:=w={\left(w_{1},\ldots \;,w_{K}\right)}^{T}\in R^{K}:w_{i}=0\;\text{or}\;1,\;\text{and}\;\sum_{i=1}^{K}w_{i}=1.$$

For *projectToSimplex*, we use a similar algorithm as that proposed in (Chen and Ye, 2011), and *projectToVertex* directly projects ***v*** to the standard unit vector of the same maximum value. △t is step size in projected gradient descent method satisfying $△t\leq \frac{1}{L}$ for an *L*-smooth convex function we optimize. In practice, we set Δt=0.005 as default. We repeat 1 and 2 until the results of the two consecutive steps are the same. Then we get the solution of *U* at Step 1.

Step 2:Fix U to update α and β . We rewrite the objective function w.r.t the logistic regression coefficients *α* and β as follows.

$$\begin{array}{c} \underset{\alpha ,\beta }{\min}\;-\sum_{i\in Z}\;\text{log}\;Pr(u_{g_{i}i}=1|x_{i})-\sum_{i\in Z^{\prime }}\sum_{k=1}^{K}u_{ki}\;\text{log}\;Pr(g_{i}=k|x_{i})\;+\lambda_{2}\sum_{k=1}^{K-1}\left|\right|\beta_{k}|{|}_{1}\\ s.t.\;\;\;\text{log}\;\frac{Pr(g_{i}=k|x_{i})}{Pr(g_{i}=K|x_{i})}=\alpha_{k}+\beta_{k}^{T}x_{i},\;\forall 1\leq k\leq K-1,\forall i,\\ \;\sum_{k=1}^{K}Pr(g_{i}=k|x_{i})=1,\;\forall i. \end{array}$$

Given *U*, this optimization problem becomes the sparse logistic regression on all the cells. We use the R package glmnet to complete this step.

CALLR iterates step 1 and step 2 until convergence. In practice, we stop the algorithm when the results of the two consecutive steps become very close. We put the whole computation process in Algorithm 1.

**Algorithm 1 CALLR: Cell Annotation using Laplacian and Logistic Regression**

**Input:**

 *X*: scRNA-seq matrix; *Z*: index set of the annotated cells;

 $y_{Z}$: labels of the annotated cells;

 *K*: number of clusters given by the annotated dataset;

 *L*: Laplacian matrix constructed from all the cells;

 △t: step size in the descent step; $\mu =\frac{1}{\lambda_{1}}$: parameter;

**Output:**  y: cell labels;

1.
$P\leftarrow \mathrm{SparseLogisticRegression}(X_{Z},y_{Z})$

2.
$U^{\mathrm{old}}=U^{0}=0$

3.
$U^{\mathrm{new}}=\mathrm{rand}(0,1),\;u_{i}^{\mathrm{new}}\leftarrow \mathrm{projectToSimplex}(u_{i}^{\mathrm{new}}),\;\forall i\in Z,\;u_{g_{i}i}^{\mathrm{new}}=1,\;\forall j\neq g_{i},\;u_{ji}^{\mathrm{new}}=0$

4. **while**  $||U^{\mathrm{new}}-U^{\mathrm{old}}||>\epsilon_{1}$  **do**
5.
$U^{\mathrm{old}}\leftarrow U^{\mathrm{new}},\;U^{1}\leftarrow U^{\mathrm{new}},\;N=1$

6.  **while**  $||U^{N}-U^{N-1}||>\epsilon_{2}$  **do**
7.   $\tilde{U}\leftarrow U^{N}+△t(-LU^{N}+\mu \;\text{log}\;P)$
8.   **for**  j=1:n  **do**
9.    $u_{j}^{N+1}\leftarrow \mathrm{projectToSimplex}({\tilde{u}}_{j})$
10.    $u_{j}^{N+1}\leftarrow \mathrm{projectToVertex}({\tilde{u}}_{j})$
11.   N←N+1
12.  $U^{\mathrm{new}}\leftarrow U^{N}$
13.  **for**  i=1:n  **do**
14.   $y(i)\leftarrow \mathrm{which}[u_{i}^{N}==1]$
15.  P←SparseLogisticRegression(X,y)
16. **return *y***

For Algorithm 1, besides the outer iterations for alternately updating *U* and *β*, both steps include inner iterations. Step 1 involves the gradient descent step, which requires $O(Kn^{2})$ operations, and the projection and thresholding step, which requires $O(K^{2}n)$ operations. As *K *<* n*, the complexity of this step can be written as $N_{1}\times O(Kn^{2})$, where *N*_1_ is the number of inner iterations. Step 2 implements glmnet, which includes a so-called partial Newton algorithm and the coordinate descent step (Friedman *et al.*, 2010). This step is of computational complexity $N_{2}\times (O(\mathrm{Knp})+N_{3}\times O(\mathrm{Knp}))$ (Yuan *et al.*, 2012), where *N*_2_, *N*_3_ are the number of iterations for step 2 and coordinate descent within step 2. For the space complexity, step 1 requires the space of $O(n^{2}+nK)$, and step 2 requires the storage of $O(K^{2}np)$.

### 2.3 Preparation of the labeled cells

In the proposed method CALLR, we assume that we have known a few number of annotated cells. These cells can be labeled manually as usually do (Zhang *et al.*, 2019b), and they can also be selected with some state of the art computational methods. Currently, we apply the scoring technique developed in Garnett (Pliner *et al.*, 2019) to select the representative cells. The scoring framework consists of 3 steps. First, the term frequency-inverse document frequency (TF-IDF) matrix is calculated, which is defined by

$$T_{i,j}=\frac{X_{i,j}}{\sum_{i=1}^{m}X_{i,j}}\times (1+\frac{n}{\sum_{j=1}^{n}X_{i,j}}),$$

where $X_{i,j}$ is the normalized gene expression matrix defined above. Then we assign a cutoff *C_i_* of each gene

$$C_{i}=0.25q_{i},$$

where *q_i_* is the 95th percentile of *T* for gene *i*. Any value $T_{i,j}$ below *C_i_* will be set to 0. Finally, we define the marker score $S_{c,j}$ for cell type *c* and cell *j* as

$$S_{c,j}=\sum_{k\in G_{c}}T_{k,j},$$

where *G_c_* is the list of marker genes for cell type *c*. In our example, cells in the 85th percentile and above for marker score *S* in only one cell type are chosen as representatives for that type. More details can be found in (Pliner *et al.*, 2019).

## 3 Results

In this section, we evaluate CALLR using the real-world datasets and present the results compared to other models.

### 3.1 Datasets

We downloaded 10 publicly available scRNA-seq datasets, and they are summarized in Table 1. We mainly chose the data generated using 10X, which can provide high-throughput data efficiently. Datasets of five mouse organs’ scRNA-seq from Tabula Muris generated using 10X were selected, which include bladder, kidney, lung, marrow and tongue (Tabula Muris Consortium *et al.*, 2018). For the dataset ‘Lung’, depending on the marker file used in Garnett (Pliner *et al.*, 2019), we selected four cell types. ‘Baron’ is a scRNA-seq dataset for human pancreatic islets (Baron *et al.*, 2016). We selected donor one of the four donors in this study. The cells were sequenced using inDrop. Two datasets of Peripheral Blood Mononuclear Cells (PBMC) are ‘PBMC10X’ and ‘PBMCSeqWell’, which were generated using 10X and SeqWell (Butler *et al.*, 2018; Gierahn *et al.*, 2017). ‘PBMC10X’ originally includes 2638 cells from 8 cell types. According to the marker file used in Garnett (Pliner *et al.*, 2019), we combined four types of them into two, and took six types finally. ‘Chen’ is a large dataset consisting of 23 284 genes and 14 437 cells in 47 cell types (Chen *et al.*, 2017). All the cells in these datasets have their true annotated labels.

**Table 1:** Summary of the 10 real datasets

Data/references	Protocol	$N_{\mathrm{gene}}\times N_{\mathrm{cell}}$	Cell types	Tissues
Baron (Baron *et al.*, 2016)	InDrop	20 125 × 1937	14	Pancreatic islets
Bladder (Consortium *et al.*, 2018)	10X	23 433 × 2500	4	Bladder
Chen (Chen *et al.*, 2017)	Drop-seq	23 284 × 14 437	47	Hypothalamus
Kidney (Consortium *et al.*, 2018)	10X	23 433 × 2781	8	Kidney
Lung (Consortium *et al.*, 2018)	10X	23 433 × 835	4	Lung
Marrow (Consortium *et al.*, 2018)	10X	23 433 × 1732	14	Marrow
PBMC10X (Butler *et al.*, 2018)	10X	32 738 × 2638	6	Blood
PBMCSeqWell (Gierahn *et al.*, 2017)	SeqWell	6173 × 3694	6	Blood
Seger (Segerstolpe et al., 2016)	Smart-Seq	25 525 × 1099	9	Pancreatic islet
Tongue (Tabula Muris Consortium *et al.*, 2018)	10X	23 433 × 7538	3	Tongue

### 3.2 Cell-type annotation results

As CALLR is a semi-supervised learning method, it can give the exact labels for all the unannotated cells. We compared CALLR with both (semi-)supervised learning methods and unsupervised learning methods. For (semi-)supervised methods, as the problem is multi-class classification, we used accuracy to measure their performance. For both unsupervised and supervised learning methods, we used the criteria NMI and ARI to measure their performance. Adjusted Rand Index (ARI) and Normalized Mutual Information (NMI) are two widely used criteria to measure the performance of clustering. They measure the similarity between two distinct partitions (one corresponding to the true clusters in our case) over a same dataset. Suppose there are two sets of clusters $C_{A}$ and $C_{B}$ for partitions *A* and *B* over the same dataset containing *n* data points. Let $|C_{A}|=I$ and $|C_{B}|=J,\;C_{A}=\left\{C_{A1},C_{A2},\ldots ,C_{AI}\right\}$ and $C_{B}=\left\{C_{B1},C_{B2},\ldots ,C_{BJ}\right\}$. Let *n_ij_* be the number of entries that belong to both *C_Ai_* and *C_Bj_*, that is, $n_{ij}=\left|C_{Ai}\cap C_{Bj}\right|$. ARI is given as:

$$\mathrm{ARI}=\frac{RI-E(RI)}{\max(RI)-E(RI)},$$

where Rand Index (RI) is defined as:

$$RI=\sum_{i=1}^{I}\sum_{j=1}^{J}\left(\begin{array}{c} n_{ij}\\ 2 \end{array}\right)/\left(\begin{array}{c} n\\ 2 \end{array}\right).$$

NMI is defined as:

$$\mathrm{NMI}=\frac{2I(C_{A},C_{B})}{H(C_{A})+H(C_{B})},$$

where

$$\begin{array}{c} I(C_{A},C_{B})=\sum_{i,j}\frac{n_{ij}}{n}\;\text{log}\;\frac{nn_{ij}}{\left|C_{Ai}||C_{Bj}\right|},\\ H(C_{A})=-\sum_{i}\frac{\left|C_{Ai}\right|}{n}\;\text{log}\;\frac{\left|C_{Ai}\right|}{n}, \end{array}$$

and $H(C_{B})$ is defined similarly. For the (semi-)supervised learning methods, we considered sparse logistic regression in R package glmnet (Friedman *et al.*, 2010) and multiclass graph-based MBO method (Garcia-Cardona *et al.*, 2014), where sparse logistic regression is a popular supervised learning method, while MBO is a semi-supervised learning method. We also considered a deep learning method ‘ItClust’, which is a transfer learning algorithm with neural network and utilizes external well-annotated source data to better label the target data (Hu *et al.*, 2020). We took the annotated cells as the source data, and labeled the remaining cells. We randomly selected 5% of the cells with their true labels as the annotated subset, and ran the (semi-)supervised algorithms. For the unsupervised clustering methods, we considered SIMLR (Wang *et al.*, 2017), Seurat (Butler *et al.*, 2018), and SAME (Huh *et al.*, 2020). We selected SIMLR and Seurat because both are graph-based clustering methods, which have some similarities with spectral clustering. Furthermore, SIMLR integrates different kernel-based similarities to visualize and cluster the cells. Seurat performs clustering using different algorithms such as the Louvain algorithm (Blondel *et al.*, 2008), Smart Local Moving (SLM) algorithm (Waltman and van Eck, 2013), and Leiden algorithm (Traag *et al.*, 2019) to optimize the standard modularity function for the shared nearest neighbor graph, which is constructed from the *k*-nearest neighbor graph using Jaccard index. We applied the default method ‘Louvain algorithm’. SAME aggregates the clustering results from multiple methods via mixture model ensemble, thus it owns the advantages of various methods. Here, SAME aggregated the results from SIMLR, Seurat and tSNE + *k*-means (first do tSNE, then *k*-means clustering). For these methods, we directly used the R packages: SIMLR, Seurat, and SAME.

**Fig. 1.**
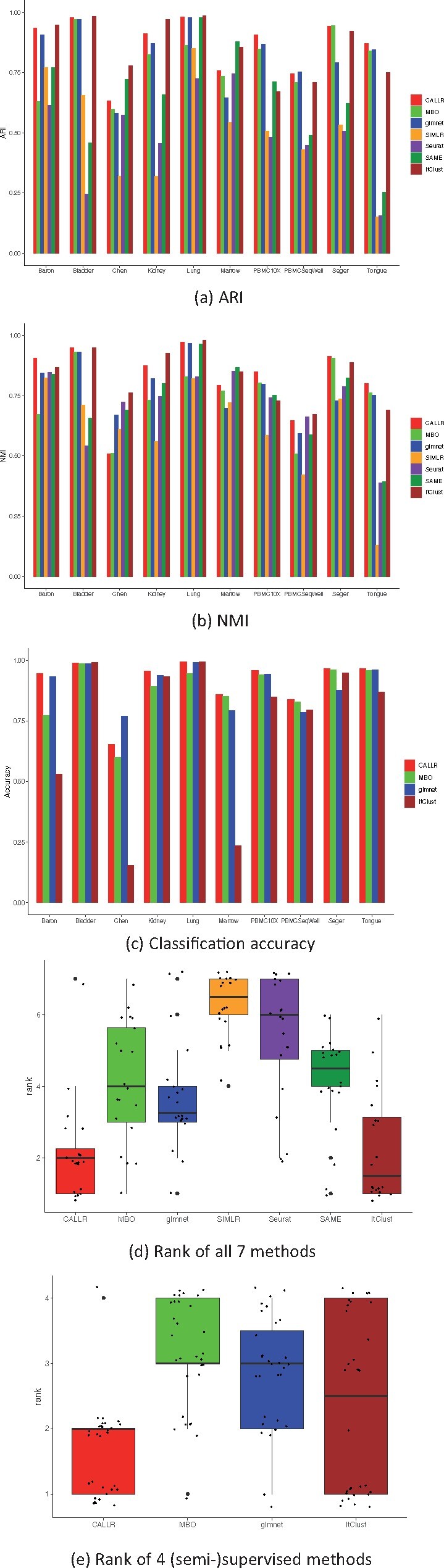
Performance comparison on 10 benchmark datasets. (**a**) ARI. (**b**) NMI. (**c**) Classification accuracy. (**d**) Rank of all seven methods. (**e**) Rank of four (semi-)supervised methods

We first compared CALLR with the clustering methods. Since the results from sparse logistic regression, MBO and ItClust can be taken as clusters, we also measured these three methods using ARI and NMI. All the results are shown in Figure 1a and b. According to both ARI and NMI, CALLR performs the best or second best in almost all datasets. Only ItClust performs slightly better than CALLR throughout 10 datasets. As ItClust is a deep learning framework, it may capture more effective details in some specific data than CALLR does. Figure 1d plots the boxplot for the performance of the seven methods. We ranked each model according ARI and NMI for 10 datasets. There are a total of 20 ranks for each model. The boxplot shows each model’s ranks across all datasets. Lower rank represents better performance (one is the best and seven is the worst). It is clear that CALLR performs more stable than ItClust. We note that CALLR does not perform well in dataset ‘Chen’. This is because when we applied CALLR to ‘Chen’, we divided the dataset into several subsets to separately get the final labels based on the same labeled subset due to the large size of this dataset. This also motivates us to develop faster algorithms for our model.

**Fig. 2.**
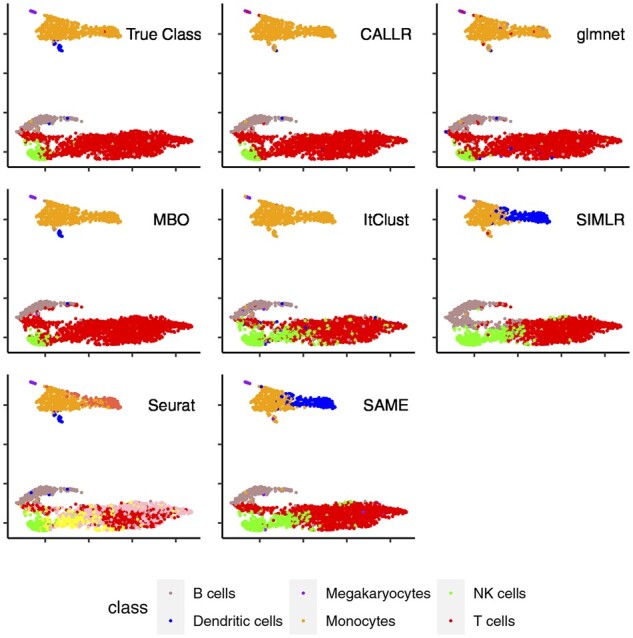
Visualization of the cells in ‘PBMC10X’

We then compared CALLR with sparse logistic regression, MBO and ItClust. All of these four methods can give exact label for each cell. The accuracy of the classification is shown in Figure 1c. In both ‘Bladder’ and ‘Lung’, four methods perform similarly because these two datasets only contain four types of cells. CALLR and ItClust achieve a little higher accuracy. For the dataset ‘Chen’, again, due to the separate subset labeling, CALLR does not perform well. For the remaining seven datasets, CALLR performs significantly better than the other three methods. In ‘Baron’, ‘Chen’ and ‘Marrow’, CALLR performs much better than ItClust because they are of more cell types. It indicates that CALLR has great adaptability to deal with large datasets with complex cell types compared to ItClust. We also show the boxplot of CALLR, MBO, glmnet and ItClust for all indexes. The results are shown in Figure 1e. CALLR has an outstanding and robust performance compared to the other three.

To clearly see the differences of these compared methods, we visualized the cells in a 2D space using umap (Becht *et al.*, 2019). We put the dataset ‘PBMC10X’ as the example. The results are shown in Figure 2. It is clear that (semi-)supervised methods perform much better than pure clustering. For (semi-)supervised methods, CALLR assigns more cells to their types correctly. To be specific, CALLR can successfully separate NK cells and T cells while other methods fail to distinguish some cells from these two types.

**Fig. 3.**
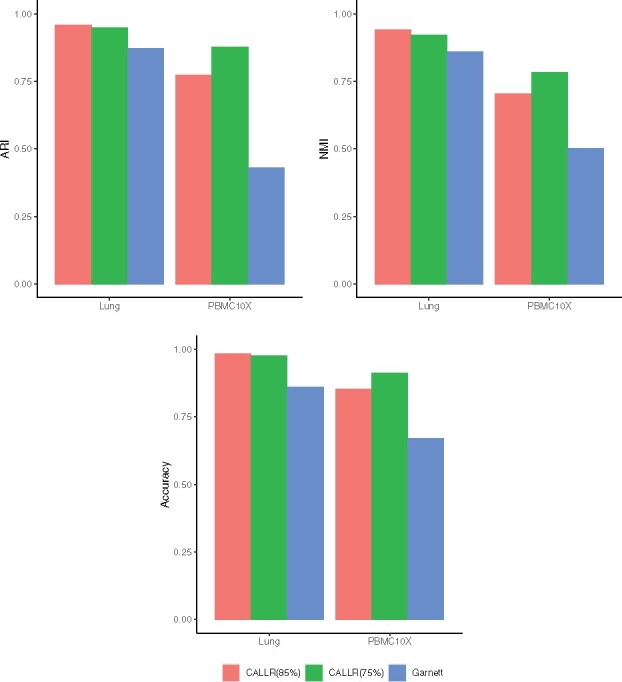
Cell-type annotation with the labeled cells determined by marker genes. ‘CALLR’ uses two thresholds the 75th and 85th percentile for representative cell selection

We further compared CALLR with Garnett, which uses marker genes to first determine the types of a small set of cells. We downloaded the marker gene files of ‘Lung’ and ‘PBMC10X’ directly from the Supplementary Materials of Garnett, and applied the same scoring technique developed in Garnett to first determine the labels of a small number of cells. In Garnett, cells having aggregated marker score greater than the 75th percentile in only one cell type are chosen as good representatives. For CALLR, we used two thresholds: the 75th percentile and the 85th percentile. The results are shown in Figure 3. In both cases, CALLR have much better performance than Garnett. For ‘Lung’, CALLR gave similar performance in both cases. For ‘PBMC10X’, more known labeled cells gave better performance, which is consistent with our intuition. All the results show that even with fewer known labeled cells, CALLR greatly improves sparse logistic regression.

We conducted the experiments in a Laptop with CPU 3.1 GHz Intel Core i5 and memory 8 GB 2133 MHz LPDDR3 to check the computational time. When the cell size is of 835 (dataset ‘Lung’), it took 3 min. When the cell size is of 2500 (dataset ‘Bladder’), it took 15 min. And when the sample size is of 7538 (dataset ‘Tongue’), it took about 2 h and a half. When the sample size is very large (dataset ‘Chen’), we divided the cells into several groups correspondingly, and ran the algorithm separately to cluster each group. This procedure can save time and space, but may lose some annotation accuracy.

### 3.3 Effect of the number of labeled cells

We did experiments to investigate the relationship between the labeled cells’ size and the performance of annotation. We denoted the ratio of the size of labeled cells to the total sample size as $r=\frac{n^{\prime }}{n}$. Here we show the results on the ‘Lung’ data matrix.

**Fig. 4.**
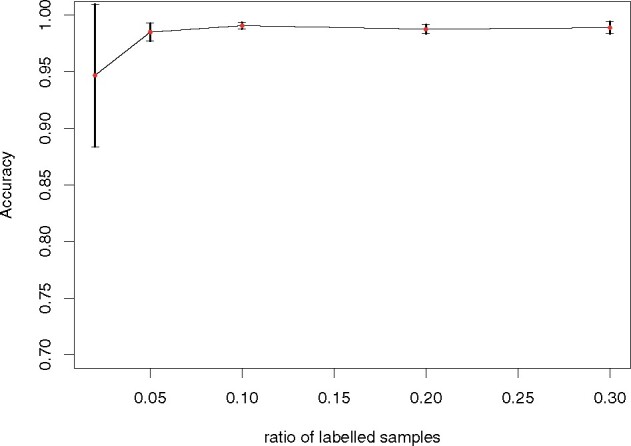
Classification accuracy for different ratios of labeled cells

We let r=0.02,0.05,0.1,0.2,0.3 to select the labeled cells randomly. For each value of *r*, we repeated the experiments for 10 times and calculated the accuracy of classification to the cell types. We recorded the accuracy means and standard deviations. The result is shown in Figure 4. When *r* is very small, the results highly depend on the number of labeled cells. When *r *>* *0.05, the results become very stable. This shows that CALLR needs only a few labeled cells, and they can help improve the annotation greatly. In the vast majority of cases on different datasets, we have 0.05<r<0.3 can give reliable results.

### 3.4 Parameter selection

The optimization problem contains two tuning parameters: *λ*_1_ for balancing the effect of logistic regression term and the spectral clustering term, and *λ*_2_ for regularization in sparse logistic regression. For *λ*_2_, it can be selected in the dataset with labels using leave-one-out cross validation. Based on the empirical results, in practice, we directly set $\lambda_{2}=0.004$.

The selection of *λ*_1_ is equivalent to selecting *μ* as previously mentioned in step 1 of Algorithm 1, where the two terms in the objective function are corresponding to logistic regression and spectral clustering, respectively. There should be high consistency between the clusters obtained using both methods separately, and the number of borderline cells that affect the final classification results in logistic regression should be small. Thus the model should be robust to the choice of the parameters. In our setting, both terms are linear functions of the cell number *n*. The log-likelihood is a sum of about *n* terms, and the trace term is a sum of about *nK_nn_* terms, where *K_nn_* is the number of neighbors in the construction of the similarity graph. Thus to balance these two terms will not highly depend on *n*. In practice, we varied the parameter in different datasets to see the performance, and finally took μ=0.3 as the default.

**Fig. 5.**
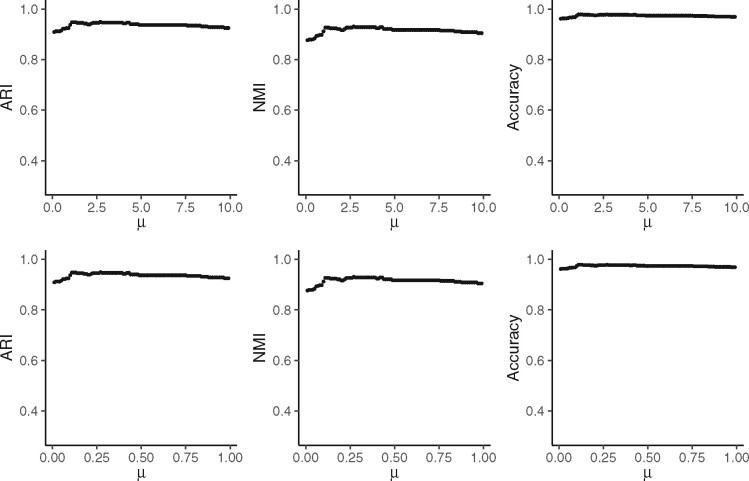
Performance of CALLR for different values of *μ* on the dataset ‘Lung’

We took the dataset ‘Lung’ as an example to show the results for different values of parameter *μ*. First, we set μ=0.1,0.2,0.3,…,10 with step size 0.1 and ran the algorithm to investigate the variation of clustering performance. As we can see in the first row of Figure 5, for either NMI, ARI or accuracy, the best performance happens when μ∈[0,1], and as *μ* goes larger, the performance of CALLR is very stable, though it becomes a little worse. We further checked out the effect of *μ* more closely. Let μ=0.01,0.02,0.03,…,1 with step size 0.01, and ran the algorithm. The result is shown in the second row. Either NMI, ARI or accuracy reaches their highest value when *μ* is around 0.3, and the value of these measures changes quite small.

## 4 Discussion

We presented CALLR, a semi-supervised learning framework, to annotate the cell types. It learns the labels of the unannotated cells from the log-likelihood function and Laplacian matrix. Based on a small number of labeled cells and the similarity graph between different cells, it can predict the probabilities of those unlabeled cells being in a particular type. The resulting information alternatively helps the clustering. As a result, CALLR combines the advantages of sparse logistic regression and spectral clustering to annotate each cell more accurately. For the representative cells of each type, with information of marker genes, we can conduct the selection using the existing data-driven approaches, which makes it easier to use our proposed method. We applied alternating optimization method and projected gradient descent method to solve the proposed optimization model. Such algorithms reduce the computational complexity of binary optimization, and thus improve the computational efficiency and capacity of the model. Furthermore, analyzing the effect of the labeled cells’ size on the annotation results shows the robustness of CALLR. The performance of the method is stable when parameter changes or labeled subset varies.

Results across 10 real datasets show that CALLR provides more accurate and robust results. For either NMI, ARI or accuracy as assessment criteria, the performance of CALLR is the best compared to the traditional (semi-)supervised methods. And it is competitive compared to the up-to-date deep learning method ‘ItClust’ with more stable performance. According to NMI and ARI, it outperforms each single compared clustering method. And it outperforms SAME in 8 of the 10 datasets, where SAME integrates the advantages of various current clustering methods. We note that in CALLR, the number of cell types depends on the annotated cells, which is pre-specified. It may not detect the rare cell types since it is difficult to find the representative cells at the first stage due to their small sample size. However, some clustering methods, such as those in Seurat, learn the number of cell types automatically, which may help determine the number of cell types in advance. Taking advantages of such clustering methods and the increasing number of marker genes to label a number of reliable representative cells is of great importance, and is left as one of our future work.

In our current formulation and experiments, we only used one Gaussian kernel function to construct the adjacency matrix of all the cells, which is based on the pairwise Euclidean distance. As there are many kernel-based similarity fusion methods developed, we may integrate more similarity measures to construct the adjacency matrix, which have shown better performance, such as SIMLR. At the same time, dimension reduction methods can also be applied before measuring the similarities between pairwise cells.

The implementation of CALLR is based on general and rigorous theories behind logistic regression, spectral clustering and graph-based Merriman–Bence–Osher scheme. Thus, it is a useful classification framework not only for single cells but also for other fields, such as pattern recognition and image processing.

## Acknowledgements

The authors thank Mr. Jinhu Li at Peking University and Mr. Biao Zhang at Fudan University for their helpful discussions related to the project.

## Funding

This work was supported, in part, by Science and Technology Commission of Shanghai Municipality [No. 20ZR1407700] and Key Program of National Natural Science Foundation of China under Grant [No. 61932008].

*Conflict of Interest*: none declared.
